# Survey Research Among Neurosurgeons: A Bibliometric Review of the Characteristics, Quality, and Citation Predictors of the Top 50 Most-Influential Publications in the Neurosurgical Literature

**DOI:** 10.7759/cureus.64785

**Published:** 2024-07-17

**Authors:** Abdulhakim B Jamjoom, Abdulhadi Y Gahtani, Jude M Jamjoom, Belal M Sharab, Omar M Jamjoom, Moajeb T AlZahrani

**Affiliations:** 1 Section of Neurosurgery, King Saud bin Abdulaziz University for Health Sciences, Jeddah, SAU; 2 Department of Medical Education, Alfaisal University College of Medicine, Riyadh, SAU; 3 Department of Medical Education, Ankara Yildirim Beyazit University, Ankara, TUR; 4 Department of Pharmaceutical Care Services, King Abdulaziz Medical City, Western Region, Jeddah, SAU; 5 Section of Neurosurgery, King Saud bin Abdulaziz University for Health Sciences College of Medicine, Jeddah, SAU

**Keywords:** citation rates, pubmed, publication trends, bibliometrics, spine journals, neurosurgical journals, survey questionnaire

## Abstract

Survey research enables the gathering of information on individual perspectives in a large cohort. It can be epidemiological, attitude or knowledge focussed. Assessment of survey studies sampling neurosurgeons is currently lacking in the literature. This study aimed to highlight the characteristics, quality, and citation predictors of the most influential survey research studies published in the neurosurgical literature. Using PubMed and Google Scholar, the 50 most cited survey research publications were identified and reviewed. Data relating to the characteristics of the articles, participants and questionnaires were retrieved. The studies’ quality and citation patterns were assessed. The median articles' age and publishing journal impact factor (IF) were 15.5 years and 2.82, respectively. Thirty-two (64%) articles were first authored by researchers from the USA while 28(56%) studies were focussed on specific disease management. The median number of participants and response rates were 222 and 51%, respectively. A full version of the questionnaire was provided in 18 (36%) articles. Only four (8%) articles reported validation of the questionnaire. The overall quality of reporting of the surveys was considered fair (based on good grading in five parameters, fair grading in one parameter, and poor grading in four parameters). The median citation number was 111. The citation analysis showed that the participant number, article age (≥15.5 years), and questionnaire category (surgical complications) were significant predictors of citation numbers. The citation rates were not influenced by the response rates or the journal’s IF. In conclusion, high-impact survey publications in the neurosurgical literature were moderately cited and of fair quality. Their citation numbers were not affected by response rates but were positively influenced by the publication age, number of participants, and by novel data or the questions raised in the survey category. Surveys are valuable forms of research that require extensive planning, time, and effort in order to produce meaningful results. Increasing awareness of the factors that could affect citations may be useful to those who wish to undertake survey research

## Introduction and background

Surveys are research methods in which information is typically collected by asking a subset of people questions on a specific topic and generalizing the results to a larger population [1,2]. They are widely used in many specialities, but mostly in public health, politics, eth­ics, and education [1,2]. Surveys are particularly valuable in studies that need patients or clinicians to self-report their experiences, satisfaction, and attitudes towards concepts that are difficult to measure using alternative approaches [1,2]. They are useful at the beginning of explor­atory studies and can be the basis for going on to the next levels of evidence [1,2]. Surveys are generally considered low-cost research tools that are easy to apply [2]. However, to be reliable, they require good planning and construction as well as clear reporting so readers can judge the strengths and weaknesses of the study as well as the generalizability of the results [1-3]. The number of surveys published in the medical literature has increased in recent years [1,3]. Furthermore, assessing the quality of survey research is a topic of current interest in the literature [1-5]. Most of the published reviews stated that the quality of the reporting of surveys was either poor [1,3] or moderate [2,4,5]. The issues raised included weak designs of questionnaires, lack of validation and reliability tests of the instruments used, low response rates, shortage of information on the representativeness of the samples, and deficiency of explanations on how missing data was handled [1-5]. Recent guidelines for clinical survey research emphasized the need to reduce potential sources of bias by using validated questions, pre-testing the questionnaires, and enhancing response rates by using incentives or reminders [2,4].

The number of citations a published article receives is arguably one of the most important indicators of its impact and clinical weight. Identification of the predictors of citations is valuable for researchers to enhance the impact of their work. It is a topic that has been the subject of numerous studies in recent years. Most publications on the matter concentrated on determining the predictors of citations in articles relating to certain medical specialties, disease processes, peer-reviewed journals, or a specific research methodology [6,7]. Analysis of the citation patterns of survey research remains limited in the literature. Furthermore, assessments of survey studies sampling neurosurgeons are also lacking. The purpose of this study is to identify and review the most-cited survey research studies that were published in the neurosurgical literature. The review aimed to highlight the characteristics and quality of survey research studies that sampled neurosurgeons and determine the factors that affect the citation numbers of the top 50 most-influential studies on the subject.

## Review

Methods

Search Strategy

No ethical approval was necessary by our institutions as the study was based on data obtained from open-access sources. The PubMed database was searched on 1st October 2023 for suitable articles using the following combinations: [Title] “survey” AND [Journal] “individual neurosurgical and spine journals by name”. The list of journals searched, and the number of screened articles is shown in Table 1. The search yielded a total of 921 publications in 30 neurosurgical and spine journals which will be referred to as neurosurgical journals in this study.

**Table 1:** List of the searched neurosurgical journals showing numbers of the screened and selected articles

Searched journals	Screened articles number	Selected most cited studies number
Spine	100	7
Journal of Neurosurgery	66	7
World Neurosurgery	136	6
Neurosurgery	45	6
Acta Neurochirurgica	69	3
Pediatric Neurosurgery	14	3
Child's Nervous System	36	3
Clinical Neurology and Neurosurgery	32	2
Spine Journal	22	2
Journal of Neurosurgery Pediatrics	22	2
Stereotactic and Functional Neurosurgery	9	2
Journal of Neurosurgery Spine	20	2
Surgical Neurology	14	2
European Spine Journal	60	1
British Journal of Neurosurgery	39	1
Pituitary	8	1
Spinal Cord	63	0
Journal of Neurology Neurosurgery and Psychiatry	41	0
Joint Bone Spine	34	0
Neurologia Medico-Chirurgica	19	0
Neurosurgical Review	11	0
Journal of Neurosurgical Sciences	11	0
Neurosurgical Focus	10	0
Surgical Neurology International	10	0
Journal of Neurological Surgery Part B Skull Base	8	0
Journal of Neurological Surgery Part A Central European Neurosurgery	7	0
Journal of Korean Neurosurgical Society	6	0
Neurospine	3	0
Asian Journal of Neurosurgery	3	0
Clinical Neurosurgery	3	0
Total	921	50

Using Google Scholar, the citation numbers for all screened articles were documented. In view of the regular changes in the citation numbers, the findings on a single day (15th December 2023) were documented and used for analysis. The 50 most-cited articles were identified and chosen for this review. The selection was limited to surveys published in the neurosurgical journals in which the participants were surgeons, surgical residents or a combination of surgeons and non-surgeons. We excluded studies in which all participants were non-surgeons and articles that did not provide an adequate description of the survey process or other pertinent data. The selected articles are referred to as "most cited", or "most influential", or as "high-impact survey research articles sampling neurosurgeons" interchangeably in this review.

Analysis of Characteristics

Using the full articles, relevant information relating to each of the selected studies was collected by two of the authors independently and any discrepancies were resolved by consensus. Missing data was referred to as not available (NA). The extracted data was grouped into: Articles’ characteristics: publication year, publishing journal, its impact factor (IF), number of authors, number of centres, number of specialties, number of countries and first authors’ countries. The journals' IF data was obtained from an online source [8]. Participants’ characteristics: their number, population (whether individuals, groups, or centres), selection method (whether from affiliation to associations or workgroups, medical meeting attendees, panel of experts or random individuals or hospitals), response rates, specialties, and worldwide regions. Questionnaires’ characteristics: number of items on the questionnaire, subspecialties, categories (whether specific disease management, training and career, surgical complications, or methods and techniques) and the individual topics.

Analysis of Quality

The quality assessment was comparable to others [1-5] and was based on whether several parameters were clearly reported in the articles. These were: study population, sample selection methods, sample size, response rate, incomplete response rate, non-responders characteristics, the full version of the questionnaire, number of items on the questionnaire, and whether the questionnaire was validated or pretested. The quality of reporting of each parameter in ≥ 66% of articles was graded as good, in 34%-65% of articles was graded as fair and in ≤ 33% of articles was graded as poor. The quality grading was determined by two of the authors independently and any discrepancies were resolved by consensus.

Analysis of Citation Predictors

The citation predictors assessment was carried out by correlating the citation numbers for the selected studies with the various article, participant, and questionnaire characteristics. The correlation testing was done by calculating the Pearson correlation coefficient (R) using the Social Sciences Statistics website [9], and significance was determined when p ≤ 0.05. A secondary citation predictors analysis was carried out by comparing the mean citation numbers [± standard deviation (SD)] between the following subgroups: articles’ ages [≥15.5 versus (vs.) <15.5 years], journals’ IF (≥2.82 vs. <2.82), number of authors (>4 vs. ≤4), number of centres (1 vs. >1), number of specialties (1 vs. >1), number of countries (1 vs. >1), first authors’ countries (USA vs. others), participants’ numbers (≥222 vs. <222), participants’ response rates (≥51% vs. <51%), participants’ specialties (general neurosurgeons vs. others), participants’ selections (associations/groups vs. others), participants’ worldwide regions (North America vs. others), questionnaires’ number of items (≥12 vs. <12), questionnaires’ subspecialties (spine vs. others), questionnaires’ categories (management/ training and career/ complications vs. others). The median was taken as a cut-off point in the numerical parameters The statistical analysis was carried out by calculating the mean difference (MD) using the MedCalc website [10]. Significance was determined when p ≤ 0.05.

Results

The 50 most-cited survey research studies sampling neurosurgeons are summarised in Table 2 [11-60].

**Table 2:** The selected 50 high-impact studies of survey research among neurospine surgeons *Respondents only Abbreviations: NA: not available, Ref: Reference, Particip.: Participants, Neurosurg: Neurosurgery, Neurol: Neurology, Pediatr: Pediatric, Sterotact Funct: Stereotactic and Functional, SSEP: somatosensory evoked potential,  SCI: spinal cord injury, s.: spondylotic, ICP: intracranial pressure, MR: magnetic resonance, MCA: middle cerebral artery.

Rank	1^st^ Authors Year [Ref]	Journals	Particip. number	Response Rate (%)	Categories	Topics	Cites
1	Ciric I 1997 [11]	Neurosurgery	3172	82%	Complications	Transsphenoidal surgery	1345
2	Wright N 1998 [12]	Journal of Neurosurgery	847	25%	Complications	Vertebral artery injury during C1-2 fixation	628
3	Dawson E 1991 [13]	Spine	330	74%	Techniques	SSEP during spine surgery	306
4	Neo M 2008 [14]	Spine	36 groups	89%	Complications	Vertebral artery injury in cervical spine surgery	275
5	Schijman E 2004 [15]	Child's Nervous System	246	31%	Management	Chiari and syringomyelia	262
6	Härtl R 2013 [16]	World Neurosurgery	3348	20%	Techniques	Navigation in spine surgery	228
7	Sanford R 1994 [17]	Pediatric Neurosurgery	40	28%	Management	Craniopharyngioma	221
8	Haroun R 2000 [18]	Pediatric Neurosurgery	234	33%	Management	Chiari and syringomyelia	204
9	Santarius T 2008 [19]	British Journal of Neurosurgery	215	52%	Management	Chronic subdural hematoma practice survey	187
10	Laitinen L 1985 [20]	Journal of Neurosurgery	16	100%	Management	Parkinson Disease surgical targets	163
11	Krauss J 2004 [21]	Acta Neurochirurgica	82	65%	Management	Normal Pressure Hydrocephalus	160
12	Belzberg A 2004 [22]	Journal of Neurosurgery	126	39%	Management	Brachial plexus injury	154
13	Rocque B 2011 [23]	Journal of Neurosurg Pediatr	710	30%	Management	Chiari and syringomyelia	143
14	Fujibayashi S 2017 [24]	Spine	583	12.3%	Complications	Lateral interbody fusion complications	136
15	Eck J 2006 [25]	Spine	1322	23%	Management	Prednisolone in acute SCI	135
16	Cohen-Gadol A 2005 [26]	Neurosurgery	710	26%	Training & career	Residents duty hours reform	127
17	Dipaola C 2009 [27]	Spine Journal	133	86%	Management	Osteoporosis and osteomalacia	125
18	Whitehead W 2001 [28]	Pediatric Neurosurgery	129	65%	Management	Shunt infections practice survey	124
19	Tamburrini G 2008 [29]	Child's Nervous System	60	75%	Management	Sylvian fissure arachnoid cysts	123
20	McAbee J 2015 [30]	Journal of Neurosurgery	3247	24%	Training & career	Satisfaction among neurosurgeons	123
21	Abosch A 2013 [31]	Sterotact Funct Neurosurgery	146	45%	Techniques	Deep Brain Stimulation procedural steps	120
22	Cheng M 2000 [32]	Neurosurgery	986	40%	Complications	Visual loss after spine surgery	120
23	Giustina A 2011 [33]	Pituitary	73	89%	Management	Acromegaly management practices	118
24	Jhawar B 2007 [34]	Journal of Neurosurg Spine	138	68%	Complications	Wrong side and level in neurosurgery	118
25	Attenello F 2018 [35]	Journal of Neurosurgery	1643	21%	Training & career	Burnout among residents	112
26	Favre J 1996 [36]	Neurosurgery	(28 centres*)	NA	Management	Pallidotomy practice survey	109
27	Ganju A 2013 [37]	World Neurosurgery	99	53.5%	Training & career	Simulation in neurosurgical education	108
28	Auerbach J 2011 [38]	Spine	904	62%	Training & career	Musculoskeletal disorders among spine surgeons	107
29	O'Neill B 2008 [39]	Surgical Neurology	3100	30.4%	Techniques	ICP monitor placement	104
30	Kaufman H 1991 [40]	Surgical Neurology	2969	38%	Management	Care of gunshot wounds to the head	97
31	Steinbok P 2006 [41]	Journal of Neurosurgery	(105)*	NA	Management	Occult tethered cord syndrome	92
32	Uribe J 2015 [42]	European Spine Journal	77	52%	Complications	Lateral interbody fusion complications	92
33	Ondo W 2005 [43]	Sterotact Funct Neurosurgery	47	77%	Techniques	Deep Brain Stimulation placement and adjustment	87
34	Bible J 2009 [44]	Spine Journal	142	83%	Management	Bracing after spine surgery	86
35	Haines S 1991 [45]	Neurosurgery	152	63%	Management	Chiari malformation Types I and II	86
36	Rapport R 2^nd^ 1973 [46]	Journal of Neurosurgery	1354	78.6%	Management	Prophylaxis for posttraumatic epilepsy	86
37	Ghogawala Z 2007 [47]	Spine	239	38%	Management	Ventral vs. dorsal decomp in cervical s. myelopathy	81
38	Ikezaki K 1997 [48]	Clinical Neurol Neurosurg	2096	53%	Management	Haemorrhagic Moyamoya disease	79
39	Rofes A 2017 [49]	Acta Neurochirurgica	28 centres	75%	Management	Cognition in low-grade glioma	76
40	Schroeder G 2014 [50]	Spine	(84)*	NA	Management	Methylprednisolone in acute SCI	75
41	Hayashi K 2013 [51]	Clinical Neurol Neurosurg	2998	39.5%	Management	Moyamoya Disease in Japan	71
42	Kshettry V 2014 [52]	World Neurosurg	100	65%	Training & career	Laboratory dissection in neurosurgical residency	71
43	Oi S 1992 [53]	Child's Nervous System	30	50%	Management	Worldwide survey of pineal tumors	70
44	Ratliff J 2009 [54]	Journal of Neurosurg Spine	(229)*	NA	Complications	Spinal surgery complications	69
45	Hankinson T 2011 [55]	Journal of Neurosurg Pediatr	269	32%	Management	MR of diffuse intrinsic pontine glioma	69
46	Fontanella M 2020 [56]	World Neurosurgery	(446)*	NA	Training & career	Effect of Covid on neurosurgical practice	68
47	Suyama K 2014 [57]	World Neurosurgery	556	46.6%	Management	Hemicraniectomy for MCA infarction	63
48	Sakowitz O 2006 [58]	Neurosurgery	130 centres	77%	Management	Ruptured intracranial aneurysm in Germany	64
49	Stienen M 2016 [59]	Acta Neurochirurgica	(532)*	NA	Training & career	Quality of resident training in Europe	62
50	Al Khalili K 2014 [60]	World Neurosurgery	100	46%	Training & career	Neurosurgery resident selection	60

The studies’ characteristics were as follows:

Article Characteristics

**Figure 1:**
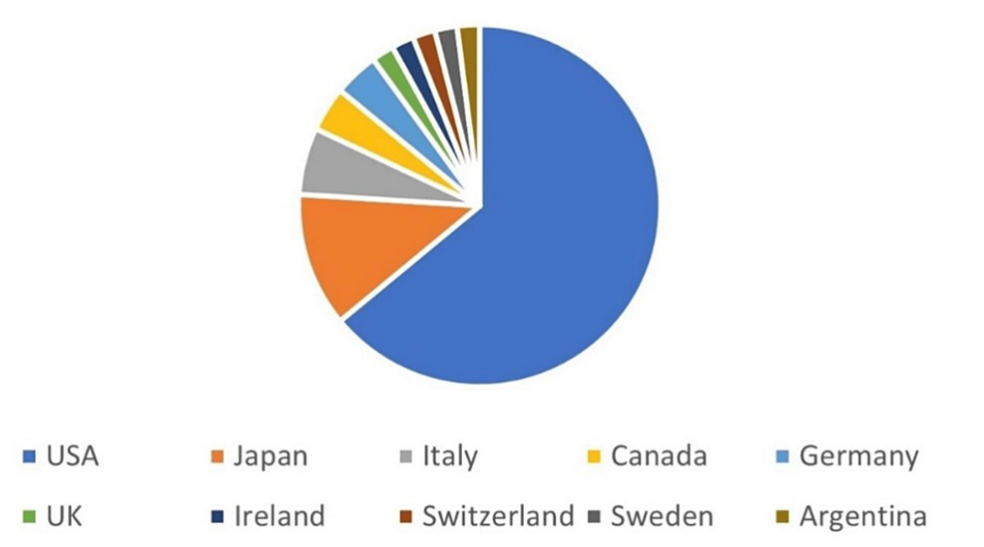
The distribution of the 50 high0impact articles according to the first authors’ countries

The median (range) publication year and articles’ age were 2007-2008 (1973-2020) and 15.5 (3-50) years respectively. The publishing journals are listed in Table 1. The most common journals were *Spine *seven (14%), *Journal of Neurosurgery* seven (14%), *World Neurosurgery* six (12%), *Neurosurgery six *(12%), *Acta Neurochirurgica* three (6%), *Pediatric Neurosurgery* 3 (6%), and *Child's Nervous System* three (6%). The median (range) journals’ IF was 2.82 (1.12- 5.41). The median (range) number of authors was four (1- 30). The median (range) number of centres was 1.5 (1- 25). The median (range) number of specialties was one (1-6). The median (range) number of countries was one (1- 9). The distribution of the articles according to the first authors’ countries is shown in Figure 1. These countries were USA: 32 (64%), Japan: six (12%), Italy: three (6%), Canada: teo (4%), Germany: two (4%), UK: one (2%), Ireland: one (2%), Switzerland: one (2%), Sweden: one (2%), and Argentina: one (2%).

Participant Characteristics

**Figure 2:**
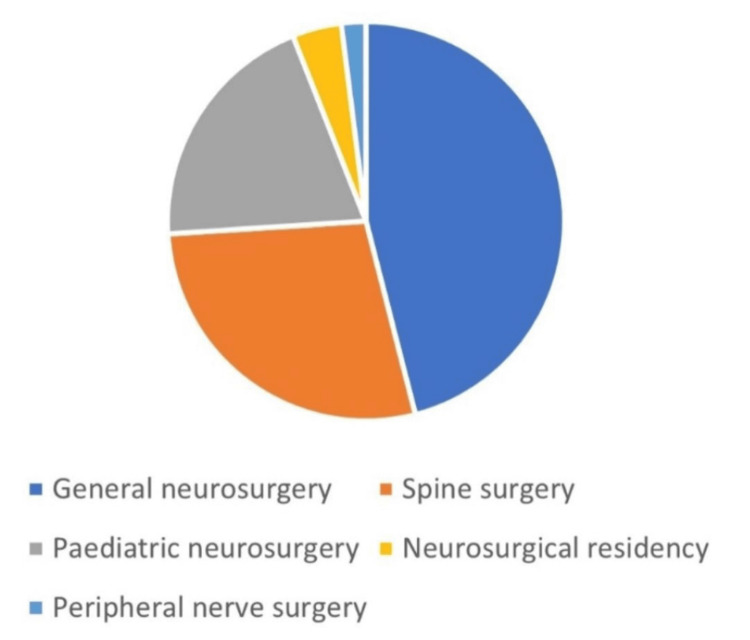
The distribution of the 50 high-impact articles according to the participants’ specialties

The median (range) number of participants and percentage response rates were 222 (28-3348) and 51% (20%-100%), respectively. The distribution of the articles according to the participants’ specialties is illustrated in Figure 2. These were general neurosurgery: 23 (46%), spine surgery: 14 (28%), paediatric neurosurgery: 10 (20%), neurosurgical residency: two (4%), and peripheral nerve surgery: one (2%).

**Figure 3:**
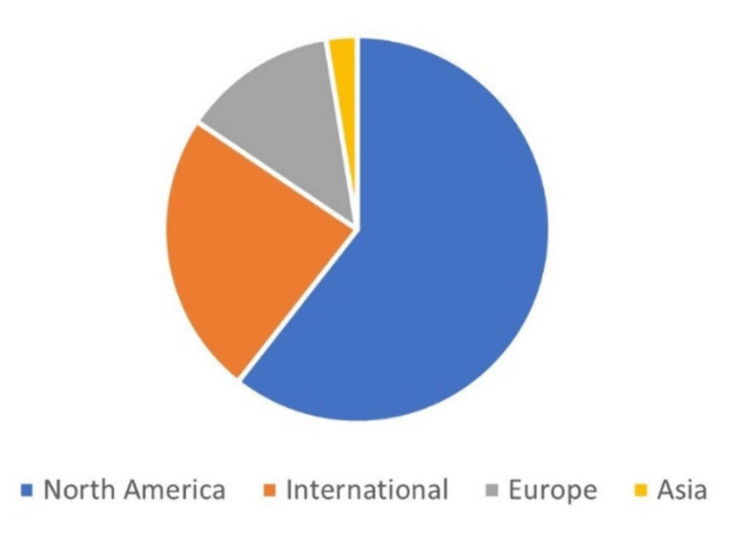
The distribution of the 50 high-impact articles according to the participants’ worldwide regions

The distribution of the articles according to the participants’ worldwide regions is shown in Figure 3. These were North America: 28 (56%), International: 11 (22%), Europe: six (12%), and Asia: five (10%).

Questionnaire Characteristics

**Figure 4:**
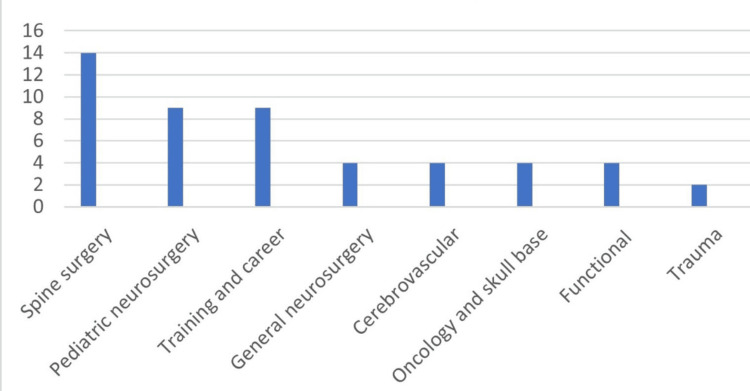
The distribution of the 50 high-impact articles according to the surveys’ subspecialties

The median (range) number of items on the survey questionnaires was 12 (2- 86). The distribution of the articles according to the questionnaires’ subspecialties is illustrated in Figure 4. These were spine surgery: 14 (28%), paediatric neurosurgery: nine (18%), training and career: nine (18%), general neurosurgery: four (8%), cerebrovascular: four (8%), oncology and skull base: four (8%), functional: four (8%), and trauma: two (4%).

**Figure 5:**
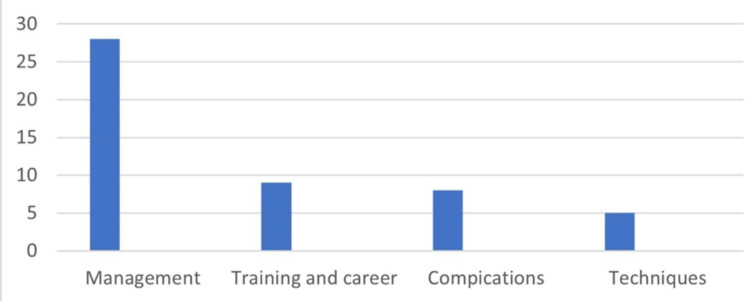
The distribution of the 50 high impact articles according to the surveys’ categories

The distribution of the articles according to the questionnaires’ categories is shown in Figure 5. These were specific disease management: 28 (56%), training and career: nine (18%), surgical complications: eight (16%), and methods and techniques: five (10%).

The most common survey topics among the 50 most-cited articles were residency training-related matters (five articles). These were duty hours reform [26], burnout and career dissatisfaction [35], the role of laboratory dissection in residency training [52], quality of residency training [59], and the residency selection process [60]. The other common topics were Chiari and syringomyelia (four articles) [15,18,23,45], the use of prednisolone in spinal cord injury (two articles) [25, 50], vertebral artery injury in cervical spine surgery (two articles) [12, 14], deep brain stimulation procedures (two articles) [31,43], complications of lateral interbody fusion (two articles) [24, 42], and Moyamoya disease (two articles) [48,51]. A full list of the topics is shown in Table 2.

Study Quality

The quality of reporting of the study population and sample selection methods was considered good, documented in 50 (100%) articles. The participants were individuals in 46 (92%) articles and centres or groups in four (8%) articles [14,36,49,58]. They were selected from associations (and working groups) in 27 (54%) articles [12,13,15-19,23-25,28,30,32, 33,35, 38,41,42,45,47-50,52-56], from random individuals or hospitals in 17 (34%) articles [11,14,21,22,26,29,34,37,39,40,46,51,54, 57-60], from meeting attendees in three (6%) articles [27,36,44], and from a panel of experts in three (6%) articles [20,31,43]. The quality of reporting of the sample size and response rates was considered good, documented in 44 (88%) articles. Six (12%) articles [36,41,50,54,56,59] reported the numbers of responders only and were not included in the calculations of the total number of participants or the response rates. The quality of reporting of the questionnaires’ number of items was considered good, documented in all articles but three [13,48,51]. The quality of reporting of the full version of the questionnaire was considered fair, documented in 18 (36%) articles [16,21,23,27,30,32, 33,35,37,42,43,45,46, 49,50, 52,55,60]. The quality of reporting of the following data was considered poor: the non-respondents characteristics, documented in two (4%) articles [19,47], the incomplete response rates (not documented in any article), the questionnaires’ validation, documented in four (8%) articles [30,34,35, 38], and the questionnaires’ pretesting, documented in one (2%) article [40]. The overall quality of reporting of the surveys was considered fair (based on good grading in five parameters, fair grading in one parameter, and poor grading in four parameters).

Study Citation Predictors

The median (range) and mean (±SD) article citation numbers were 110.5 (60- 1345) and 155.8 (± 194.6) cites, respectively. Table 3 and Table 4 summarize the correlation and secondary analysis findings between the citation numbers and the various characteristics of the selected articles. The correlation analysis showed a significant association between citation numbers and participant number (p=0.0261). The secondary analysis demonstrated significantly higher mean citation numbers in articles aged ≥15.5 years (p=0.0236) and studies in which the surveys' categories were complications-related (P=0.0016). None of the other parameters, in particular the response rates and the journals IF, reached significance

**Table 3:** Summary of the primary analysis correlation findings between the citation numbers and the various characteristics for the 50 most-cited survey research publications in the neurosurgical literature

Features	R-Value	P-Value
Articles’ age in years	0.2255	0.1154
Articles’ publishing journals	0.1762	0.2209
Articles’ journals IF	0.2265	0.1137
Articles’ number of Authors	0.1436	0.3198
Articles’ number of centres	0.12	0.4065
Articles’ number of specialties	0.0878	0.5443
Articles’ number of countries	0.0913	0.5283
Articles’ first author’s countries	0.0548	0.7054
Participants’ numbers	0.3146	0.0261*
Participants’ response rates	0.0984	0.5351
Participants’ specialties	0.031	0.8308
Participants’ selection	0.0046	0.9747
Participants’ worldwide regions	0.1064	0.4621
Questionnaires’ number of items	0.1128	0.4503
Questionnaires’ subspecialties	0.0185	0.8985
Questionnaires’ categories	0.2705	0.0574

**Table 4:** Summary of the secondary analysis mean difference findings between the citation numbers and the various characteristics for the 50 most-cited survey research publications in the neurosurgical literature *Denotes significance

Feature	Variables	Number	Mean Citation Numbers (±SD)	Mean Difference	P-value
Articles’ age (years)	≥15.5	25	199.8(±265.5)	88	0.0236*
	<15.5	25	111.8(±52.8)		
Articles’ journals impact factor	≥2.82	26	190.4(±261.5)	54.7	0.1935
	<2.82	24	118.3(±59.2)		
Articles’ number of Authors	>4	24	110.8(±54.3)	78.5	0.1544
	≤4	26	197.3(±260.4)		
Articles’ number of centres	1	25	200(±266.4)	89.3	0.1053
	>1	25	111.3(±46.9)		
Articles’ number of specialties	1	40	162.9(±214.6)	35.7	0.609
	>1	10	127.2(±74.6)		
Articles’ number of countries	1	41	163.1(±212.2)	40.8	0.5743
	>1	9	122.3(±73)		
Articles’ first authors countries	USA	32	175.1(±237.5)	53.6	0.3552
	Others	18	121.5(±66)		
Participants’ numbers	≥222	22	210.3(±281.8)	87.9	0.1581
	<222	22	122.4(±53.8)		
Participants’ response rates	≥51%	20	191.8(±278.6)	46.9	0.4572
	<51%	24	144.9(±117.1)		
Participants’ specialties	General neurosurgery	23	153.9(±262)	3.500	0.9502
	Others	27	157.4(±115.3)		
Participants’ selection	Associations/Groups	27	151.3(±116.7)	9.3	0.8664
	Others	23	160.6(±256.6)		
Participants’ worldwide regions	North America	28	172.4(±252.4)	37.7	0.5022
	Others	22	134.7(±75)		
Questionnaires’ number of items	≥12	25	169.6(±251)	29	0.6232
	<12	22	140.6(±119.1)		
Questionnaires’ subspecialties	Spine surgery	14	175.9(±150.2)	28	0.6526
	Others	36	147.9(±210.8)		
Questionnaires’ categories	Management	28	118.7(±52.1)	84.3	0.1297
	Others	22	203(±284.1)		
	Training and career	9	93(±27.4)	76.4	0.2908
	Others	41	169.5(±212.4.5)		
	Complications	8	347.9(±442.5)	228.7	0.0016*
	Others	42	119.2(±57)		

Discussion

Surveys are popular among clinical researchers, including neurosurgeons. The challenges of planning, designing, and implementing questionnaire surveys are often underestimated. A good survey requires an important topic, an appropriate sample size, an acceptable tool, a good response rate, precise results, and conclusions consistent with findings [61]. A quality survey should have the smallest possible number of high-quality essential items that will interest the population. It should also provide reproducible results (reliable) and measure what it is supposed to measure (valid) [61]. Surveys can be problematic as ensuring impartial, voluntary participation is not always easy. A large questionnaire with many nondirected questions not only deters potential respondents but also makes it difficult to evaluate validity [61]. Badgering respondents is quite likely to provoke an untruthful response, simply to meet an obligation. The participants’ responses to the questions can fluctuate due to multiple confounding reasons. The questions may be leading, unclear, or display an inherent bias of the researcher [62,63]. Surveys within small communities come with added validity drawbacks. Unlike patient or public surveys that are normally anonymous, surveys among colleagues mean that the respondents and researchers are known to each other and some responses may occur simply with the aim of pleasing the researcher rather than through a true intention to participate [62,63]. All survey measures, whether quantitative or qualitative, are subject to error. The four most common areas of survey errors are coverage (selected sample is different from the surveyed population), sampling (sample size miscalculated or not truly random), measurement (answer is inaccurate or imprecise due to poor question), and non-response (responders are different for the non-responders on the question of interest) [62,63].

Over the last decade, numerous articles reviewed survey research publications from the perspectives of several specialties. These included dentistry [2], nephrology [3], anaesthesia [4], pharmacy [5], radiology [64], critical care [65], colorectal surgery [66] and plastic surgery [67]. The number of articles evaluated in this review (50) is within the range (38-199) of articles reviewed in these studies [2-5,64-67]. Our selected articles were published over 48 years (1973-2020), which is much longer than the 1-to-21-year period covered by the other reviews [2-5,64-67]. Most of our articles were relatively old (median age 15.5 years) which may account for the deficient reporting of certain aspects of data. Our articles were published in journals with a fairly good IF (median 2.82) which is similar to others [4,66]. The majority of our surveys (56%) were carried out in North America which is within the range of 41% to 59% stated by others [2-4,65]. The reporting of the research question, study population and sample selection was considered good in all articles which is in agreement with others [2,5,64]. The majority of participants (54%) were members of associations or groups which is not surprising as it is common for surveys to sample readily available groups (convenience sampling) [3,65]. Some reviewers may have applied stricter criteria and indicated that survey reporting was weak in the description of the study population in 24% [2], the eligibility criteria for the participants in 35% [3] and the characteristics of the respondents in 10% [3].

The sample size was documented in 88% of our articles which is at the upper limit of the range of 53%-88% stated for the reporting of the sample size by other reviews [2,5]. The median response rate was 51% which is within the range of 37%-66% recorded by others [4,64]. None of our articles mentioned whether there were incomplete responses. It has been observed that the reporting of missing data in survey research has improved over the years [3]. Nevertheless, it remains a weak spot that was addressed in only 1.3% to 27% of surveys [1,2,4,5,65]. Only 4% of our articles provided data relating to the characteristics of the non-respondents. Reporting descriptions of the non-respondents is another weak point in survey research that was tackled in only 7% to 11% of studies [2,4,5]. None of our articles reported the use of incentives or reminders. Incentives were used in 7% to 30% of studies [2-5] while reminders were used in 43% to 73% of surveys [3,4,65]. A recent study however concluded that the use of reminders was not associated with higher response rates [4].

The full version of the questionnaire was provided in 36% of our articles which is within the range of 13% to 61% stated in the literature [2,5,65,67]. Only 8% of studies reported that the questionnaires were validated. In the literature, the use of validated instruments has been reported in 19% to 63% of surveys [1-5,64] while the use of previously published questionnaires was reported in 7%-50% of studies [2,5,65]. Only 2% of our articles reported that the questionnaires were pretested which is relatively low compared to the range 26%-76% mentioned by others [2,4,64,65]. The majority of questionnaires in our articles were disease-specific-management-related (56%), which is in agreement with others [3, 65]. Some authors reported that the majority of the questionnaires were current practice [66], opinion-related [67], or education [64]. This could be related to differences in the categorization of the focus of the survey between the various reports.

The median citation number for the 50 most-cited survey research studies among neurosurgeons was 111 cites. This was lower than the citation number for higher levels of evidence research studies such as the top 100 glioblastoma trials (median 349 citations) [7]. Variation in citation rates according to study design and subject is well recognized in the literature [68-70]. We found that the number of participants and the age of the publication (≥15.5) were significant predictors of citation numbers. We also observed that a research question related to specific complications in a survey was also a positive predictor of citation rates. This supports the observation that the novel data or the questions raised in the survey are an important predictor of citations [4]. In this review and comparable to others, we did not establish a positive link between citation rates and the participants’ response rates [4] or the publishing journal's IF [5]. Furthermore, none of the other parameters tested in this review affected citations. These were the number of authors, centers, specialities, countries, and the first author's country, participants’ specialty, participants’ selection, participants’ worldwide regions, surveys’ number of items, and survey subspecialties.

Limitations

There are several limitations to this study which include the general limitations of bibliometric studies. The study relied on the precision of online search engines PubMed and Google Scholar. The review did not include survey research studies among neurosurgeons that were published outside the neurosurgical literature. The presence of a good number of neurosurgical journals in one country (USA) could be a possible source of bias in both publications and citations depending on the degree of network between authors, reviewers and editors within that country. The selection of the 50 most-cited studies was based on their total citations at a certain point which was likely to change relatively quickly. This could have influenced the inclusion or exclusion of a few of the lower-impact surveys. The wide duration from publication may have affected the citations of older studies. The changing trends in the reporting of surveys over the years were not addressed. There may have been errors in the data collection. There may have been discrepancies in the grouping of articles into the various categories. The quality assessment may have not been comprehensive. Not providing the full version of the questionnaire may have been due to editorial restrictions. The review did not look at mode of administration of the questionnaires. Defining the specialty and affiliation based on the first author may not reflect all authors of multi-disciplinary papers. The impact of articles using the Altmetric score of news media and social media mentions was not done.

## Conclusions

High-impact survey publications in the neurosurgical literature were relatively old, moderately cited, and of fair quality. The majority of articles were first authored by researchers from the USA and focussed on specific disease management. Their citation numbers were not affected by response rates but were positively influenced by the publication's age, number of participants, and novel data or the questions raised in the survey category. Surveys are valuable forms of research that require extensive planning, time, and effort in order to produce meaningful results. Increasing awareness of the factors that could affect citations may be useful to those who wish to undertake survey research.
